# Increased Use of Twitter at a Medical Conference: A Report and a Review of the Educational Opportunities

**DOI:** 10.2196/jmir.2144

**Published:** 2012-12-11

**Authors:** Douglas RA McKendrick, Grant P Cumming, Amanda J Lee

**Affiliations:** ^1^Consultant AnaesthetistDepartment of AnaesthesiaDr Gray's HospitalElginUnited Kingdom; ^2^Honorary Senior LecturerSchool of Medicine and DentistryUniversity of AberdeenAberdeenUnited Kingdom; ^3^Consultant Obstetrician and GynaecologistDr Gray's HospitalElginUnited Kingdom; ^4^Honorary ProfessorUniversity of the Highlands and IslandsElginUnited Kingdom; ^5^Professor of Medical StatisticsMedical Statistics Team, Division of Applied Health SciencesUniversity of AberdeenAberdeenUnited Kingdom

**Keywords:** Twitter messaging, Social Media, Conferences, Congresses, Anesthesiology

## Abstract

**Background:**

Most consider Twitter as a tool purely for social networking. However, it has been used extensively as a tool for online discussion at nonmedical and medical conferences, and the academic benefits of this tool have been reported. Most anesthetists still have yet to adopt this new educational tool. There is only one previously published report of the use of Twitter by anesthetists at an anesthetic conference. This paper extends that work.

**Objective:**

We report the uptake and growth in the use of Twitter, a microblogging tool, at an anesthetic conference and review the potential use of Twitter as an educational tool for anesthetists.

**Methods:**

A unique Twitter hashtag (#WSM12) was created and promoted by the organizers of the Winter Scientific Meeting held by The Association of Anaesthetists of Great Britain and Ireland (AAGBI) in London in January 2012. Twitter activity was compared with Twitter activity previously reported for the AAGBI Annual Conference (September 2011 in Edinburgh). All tweets posted were categorized according to the person making the tweet and the purpose for which they were being used. The categories were determined from a literature review.

**Results:**

A total of 227 tweets were posted under the #WSM12 hashtag representing a 530% increase over the previously reported anesthetic conference. Sixteen people joined the Twitter stream by using this hashtag (300% increase). Excellent agreement (κ = 0.924) was seen in the classification of tweets across the 11 categories. Delegates primarily tweeted to create and disseminate notes and learning points (55%), describe which session was attended, undertake discussions, encourage speakers, and for social reasons. In addition, the conference organizers, trade exhibitors, speakers, and anesthetists who did not attend the conference all contributed to the Twitter stream. The combined total number of followers of those who actively tweeted represented a potential audience of 3603 people.

**Conclusions:**

This report demonstrates an increase in uptake and growth in the use of Twitter at an anesthetic conference and the review illustrates the opportunities and benefits for medical education in the future.

## Introduction

Twitter [1] is a mobile microblogging and social networking service through which its subscribers can send and read small text-based messages known as *tweets*. Tweets have a message size limit of 140 characters based on the size of the Short Message Service (SMS) messages used on mobile phones at the time of Twitter’s creation in 2006. Twitter is a technology that still has to be adopted by much of the anesthetic community. Twitter is easily accessed through a number of platforms: the Twitter website, applications (apps) developed for smartphones and tablets, and through SMS from mobile phones (in certain countries). Less than half of the tweets posted are through the Twitter website; most users prefer to use mobile apps on their smartphones or tablets [2].

Although most consider Twitter primarily a method of personal communication, it is gaining traction in business and is beginning to be used in academia for many purposes, including rapid sharing and dissemination of information and for citing articles [3]. Organizers, delegates, and speakers at meetings and conferences have found tweeting to be beneficial in their own domains and as a tool for online discussion [4]. This function of Twitter is achieved by the use of a digital “backchannel,” which is a nonverbal, real-time projection of the tweet [5]. During digital backchannel use, the speaker presents in the traditional manner in the “front” area, while the audience and people distant from the meeting can communicate with one another simultaneously by using the “back” area. This use of Twitter and other social media has the potential to change the health communications space associated with conferences.

There are only a few published reports of the use of Twitter at medical conferences [5-12]. The only report in the anesthetic literature describes an attempt by a delegate to use Twitter at the Association of Anaesthetists of Great Britain and Ireland (AAGBI) Linkman conference (September 2011, Edinburgh) who failed to attract any tweets [13]. Despite this failure, a further attempt to use Twitter for the AAGBI Annual Conference (September 2011, Edinburgh) [13] was made by the same delegate, and this time some spontaneous Twitter activity was demonstrated with no involvement from the reference conference organizers.

The aim of this study was to describe the introduction and uptake of Twitter at a major anesthetic conference with prior involvement and support from the conference organizers and analyze subsequent author use and purpose.

## Methods

A hashtag (represented by the symbol “#”) acts like a metadata tag and can be used for searches of the word/phrase strings it precedes. This makes it possible to quickly and easily collate the tweets being made at a particular conference, and even certain topics at, or subdivisions of, that conference. A hashtag (#WSM12) was created by the AAGBI 6 weeks before the start of their Winter Scientific Meeting held in London in January 2012. This hashtag was actively promoted by the organizers by using their Twitter stream, on posters around the venue, and as part of a PowerPoint presentation shown before each conference session started.

All tweets containing the #WSM12 hashtag were recorded. This record was commenced when the hashtag was first advertised and continued 14 days post conference. Any tweets made under the hashtag unrelated to the conference were excluded.

The tweets were divided into 3 main sections: (1) before the congress, which included the period from December 3, 2011 (when the hashtag was first advertised) up to 8:59 am on January 18, 2012; (2) during the conference, which included all tweets posted during the conference from 9:00 am on January 18 to 5:00 pm on January 20, 2012; and (3) after the conference, which included any tweets posted under the hashtag from 5:01 pm on January 20 to 12:00 pm on February 4, 2012.

The resulting tweets were analyzed to determine who was tweeting, during which time period of the congress they were tweeting, and to categorize the purpose of each tweet. These categories were informed by a literature review [7,10,12,14] and based on purpose of the tweet, not its content. The categories were then reclassified to be more representative of an anesthetic conference and then subclassified. These methods of classification relate to the 3 main sections: (1) before the congress (Table 1); (2) during the conference (Table 2); and (3) after the conference (Table 3). If there was any doubt as to the category of the tweeter, the tweeter was contacted directly through Twitter for confirmation.

In order to assess internal reliability of the tweet classification, the content of each tweet made during the conference was independently scrutinized by 2 of the authors (DM and GC). Each observer classified each tweet into one of the 11 categories listed in Table 2 and the kappa statistic was calculated to assess internal agreement. A kappa value more than 0.75 denotes excellent agreement; a value between 0.4 and 0.75 represents fair to good agreement [15]. All analyses were performed using the SPSS version 17 (SPSS Inc, Chicago, IL, USA).

The Twitter profile of each contributor was viewed within 2 days of the end of the conference to record the number of “followers” each tweeter had at that time. Any organizations or individuals specifically mentioned in this paper have been contacted to obtain their consent to display their Twitter profile in this publication.

**Table 1:** Tweets sent *before* the January 2012 Association of Anaesthetists of Great Britain and Ireland (AAGBI) conference.

Main category	Subcategory	Definition of tweet
Tweeter	Purpose of tweet	
Organizer	Advertising	By the AAGBI only advertising the event
	Promoting	By the AAGBI only promoting key sessions to be held at the conference
Potential delegates		By anesthetists or anesthetic groups who might potentially attend the conference
	Plans	Concerning any plans being made to attend the conference
	Advertising	Actively promoting the conference
Trade		From anesthetic trade organizations, exhibitors, or their representatives
Speakers		By any speakers at the conference who promoted their session
Others		From any other people contributing to the Twitter stream

**Table 2:** Tweets sent *during* the January 2012 Association of Anaesthetists of Great Britain and Ireland (AAGBI) conference.

Main category	Subcategory	
Tweeter	Purpose of tweet	Definition of tweet
Organizer		By the AAGBI only
Trade		By anesthetic trade organizations, exhibitors, or their representatives
Speakers		By any speakers at the conference who promoted, or discussed events during, their session
Anesthetists		By delegates attending the conference; by anesthetists not attending the conference but contributing to the Twitter stream
	Notes or learning points	Posting tweets which contained gems of information from a talk or a workshop
	Discussion	Discussing matters at the conference directly with one another; posting controversial or non-learning points from a talk
	Social	Personal discussions, social events such as dinners, coffee and lunch breaks; social posts made by the organizers
	Which session am I going to?	Tweets which described the session being attended stating the name of either the talk and or the speaker
	Encouraging speakers	Tweets directed to speakers by way of encouragement or comment
	Poster	Tweets about the posters on display
	Questions	Tweets posing questions to speakers about their presentation
Others not in attendance		Tweets by people who did not attend the conference except where those tweets directly involved discussion and encouraging a speaker

**Table 3:** Tweets sent *after* the January 2012 Association of Anaesthetists of Great Britain and Ireland (AAGBI) conference.

Main category	Subcategory	Definition of tweet
Tweeter	Purpose of tweet	
All tweeters	Continue discussions	Tweets which continued discussions and/or displayed photographs or videos of talks from the conference
	Thanks	Tweets which expressed thanks to delegates, speakers or industry, or for the meeting itself
	Reflections	Tweets mentioning the potential to use tweets posted as a method of reflecting for revalidation
	Advertising future meetings	Tweets posted to advertise future meetings, symposia, or conferences
	Feedback	Tweets containing or requesting feedback for either the conference or the speakers
	Statistics	Any statistics from the meeting presented by the organizers

## Results

A total of 227 tweets were posted under the #WSM12 hashtag during the 9 weeks of the study period surrounding the AAGBI Winter Scientific Meeting in London. An additional 18 Twitter posts from 12 people were not related to the Winter Scientific Meeting and were excluded from the current analyses. All of the excluded tweets related to a meeting involving Wireless technology usage that also used the #WSM12 hashtag for a brief period, but either changed to an alternative hashtag after realizing the duplication or completed their discussion.

Sixteen people contributed to the Twitter stream by using the #WSM12 hashtag. These 16 people consisted of the organizer and the conference venue, 3 members of trade organizations, 3 delegates attending the meeting, and 2 speakers at the meeting. The remaining 6 people who joined the Twitter stream did not attend the meeting and they were either actively promoting the meeting before the conference, contributing to discussion during the meeting, or passively retweeting some of the tweets posted by attendees.

**Figure 1:**
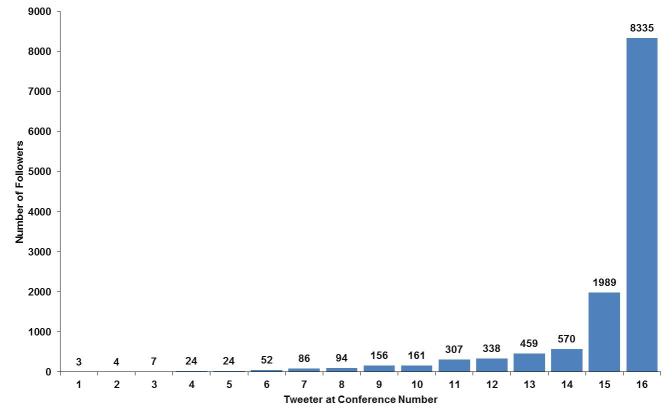
The number of people following each of the 16 people who tweeted at the January 2012 Association of Anaesthetists of Great Britain and Ireland (AAGBI) conference.

The number of followers for each of the people who contributed to the Twitter stream for the #WSM12 hashtag ranged from 3 to 8335 (Figure 1). The 16 tweeters had a combined total of 12,609 followers. Tweeters who contributed actively (access their accounts more than once a month) to the Twitter stream had 3603 followers. A retweet enables the user to share someone else’s tweet with all their own followers. A total of 32 retweets were sent during the period of the conference and, of these, 28 were sent by tweeters who were actively contributing to the Twitter stream and are also included in that group. Only 4 of the retweets were sent by people who were not actively contributing to the Twitter stream; these 4 people had a combined following of 9006 contributing to a second tier of amplification of the Twitter stream.

Of the tweets posted under the #WSM12 hashtag, 80.5% (182/227) were sent during the conference itself, 14.5% (33/227) were posted before the meeting, and 5.3% (12/227) after the conference ended. Table 4 shows the results of the internal reliability analyses on the 182 tweets sent during the conference. Excellent agreement (κ = 0.925, *P*<.001) was seen in the classification of tweets across the 11 categories with agreement of the 2 raters on 95.1% (n=173) of the 182 tweets.

**Table 4:** Interobserver reliability of the different uses of Twitter during the conference.

Category	Number of tweets coded	
	Observer 1	Observer 2
Organizers	29	28
Trade	5	5
Speakers	2	2
Notes or learning points	100	98
Discussion	8	12
Social	6	6
Which session am I going to?	21	19
Encouraging speakers	2	3
Posters	3	3
Questions	0	0
Not at congress	6	6
Total	182	182

Prior to the conference, the conference organizer posted the most tweets. Almost half (15/33, 45%) of the preconference Twitter stream was to advertise the hashtag and the conference, and 6% (2/33) to promote sessions which would be happening at the meeting. Potential delegates also advertised the meeting and the hashtag and posted 27% (9/33) of the total preconference tweets for this purpose. Another 6% of tweets (2/33) posted related to plans potential delegates were making for the meeting. A member of the anesthetic trade exhibitors also promoted the meeting in a tweet. A speaker posted a tweet promoting a session in which he would be speaking at the congress. Three other organizations used the hashtag to retweet postings advertising the meeting.

During the conference, the organizers posted 15.4% (28/182) of the tweets. They used this period of the conference to welcome delegates and advise on registration, promote trade exhibits, and to advise on various sessions and events happening at the meeting including parallel sessions, poster sessions and awards, and social events (eg, the conference dinner). The trade exhibitors at the conference posted 2.7% (5/182) of the tweets sent during the meeting to advertise their wares and special events happening at their stands. Speakers posted 1.1% (2/182) of the tweets during the meeting, with one promoting their session and the other advising of attendance and the quality of questions at their session. Anesthetists either attending the meeting, or not attending but directly contributing to the Twitter stream, posted 76.9% (140/182) of the tweets sent during the conference. Over half (100/182, 54.9%) of the tweets posted during the actual meeting were notes or learning points from various sessions at the conference. A total of 11.5% (21/182) of the tweets posted described which session people were attending, and 4.4% (8/182) of the tweets were made as a method of discussion between delegates about sessions or previously posted tweets. This discussion was joined by an anesthetist who was not able to attend the meeting. Only 3.3% (6/182) of the tweets posted related to conference social events and interactions. Another 1.6% (3/182) of the tweets related to the poster session, and 1.1% (2/182) provided support for speakers who were about to give a presentation. A total of 3.8% (7/182) of the tweets posted were sent by people not attending the meeting and not actively contributing to the Twitter stream by retweeting messages from the conference. There were no tweets posted which asked questions of any of the speakers.

There were 12 tweets posted after the conference. One-third (4/12, 33%) of the tweets were used to continue discussions started at the conference. A further 4 tweets (33%) were by way of thanks from the organizers, venue, trade, and from an anesthetist who had not attended the meeting, but appreciated the learning points generated in the Twitter stream. Another 3 tweets (25%) promoted the use of the learning points documented within the Twitter stream as a method of reflection for the purposes of revalidation. A further tweet was posted by the organizers to advertise a future meeting (1/33, 3%).

**Figure 2:**
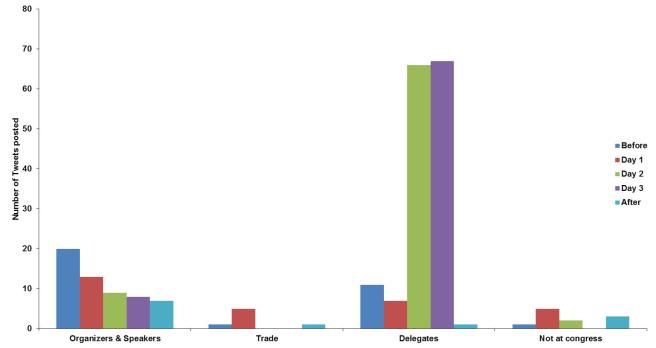
Pattern of tweets posted by the various groups of tweeters stratified by timing of the tweets. Each group is subdivided to show how many tweets they posted before the conference (Before), on which of the 3 days during the conference (day 1, day 2, and day 3), and after the meeting (After).

The pattern of tweeting by each of the main groups around the conference is shown in Figure 2. The organizers posted most of the tweets before and after the conference. The delegates started to post large numbers of tweets on the second and third day of the conference as they gained confidence in the technique (personal communication from delegates). The trade exhibition representatives tended to post their tweets advertising their products and their stands early on during the meeting.

## Discussion

This is the first report to describe the uptake and use of a Twitter stream as an integral part of the communication structure of an anesthetic conference. The total number of 227 tweets posted under the #WSM12 hashtag represents a 530% increase over the 36 tweets that “erupted” spontaneously at the 2011 AAGBI conference as reported by McKendrick [13]. Sixteen people contributed to the Twitter stream by using the #WSM12 hashtag, a 300% increase over the 4 people who spontaneously tweeted at the previous conference [13]. Although overall numbers are small, these increases become even more significant when one considers that the total attendance at this conference of 655 delegates was 16% less than the 781 delegates who attended the 2011 AAGBI conference as reported by McKendrick [13].

Although the number of people joining the Twitter stream is low (2.4% of the total number attending the conference), this is in keeping with previous studies surveying academic activity on Twitter [16]. Furthermore, this percentage may actually be representative of anesthetists who use Twitter—a recent survey of AAGBI members reported Twitter usage of 8% [17] and only 60% of Twitter account holders are active [18]. Despite these small numbers, our findings are similar to those previously described at both medical and nonmedical conferences [5-12,14]. Twitter usage has grown in a viral manner and has more than 200 million accounts with the number of daily tweets increasing by 110% during 2011 to more than 230 million tweets per day [18]. The AAGBI membership survey [17] would suggest that anesthetists have yet to embrace the exponential adoption of this new technology.

Internal reliability analyses of our classification of tweets during the conference showed a statistically significant level of intraobserver agreement that suggests that our classification system was robust.

There are many ways to use Twitter at a medical conference to enhance the experience for both those at the conference and those unable to attend. Each conference should have a Twitter profile, as should the organizer. These profiles should be promoted well in advance to maximize the number of followers, and the usefulness, of Twitter at the conference. Conference organizers should also agree on a hashtag in advance and publicize it extensively with members. The hashtag should be as short as possible to leave as much of the 140 characters for the message itself.

**Figure 3:**
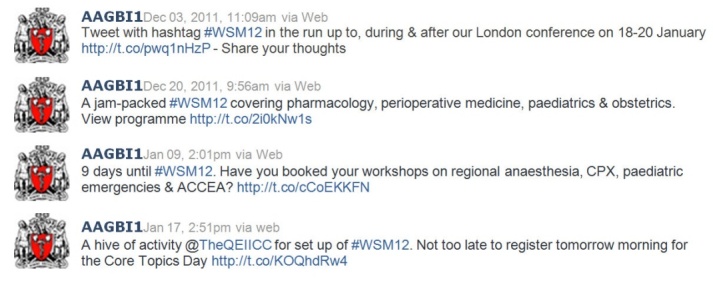
A selection of tweets posted by the conference organizers before the conference demonstrating the use of Twitter.

The conference organizers used Twitter effectively during the period before the conference to generate excitement and interest, advertise keynote presenters and workshops, and to guide delegates about registration details as illustrated in Figure 3. Attendees did not use Twitter before the conference to plan their trip, accommodation, coordinate their arrangements, or share information with colleagues [14]. Reasons could include the low number of Twitter users and Twitter naïveté, and this is an area for conference organizers to consider in the future.

**Figure 4:**
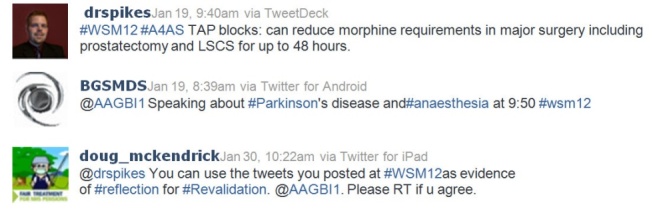
A selection of tweets posted during the January 2012 Association of Anaesthetists of Great Britain and Ireland (AAGBI) Winter Scientific Meeting. Top: The use of Twitter to post a learning point; Middle: a tweet posted by a speaker; and Bottom: part of a discussion suggesting the use of tweets as a form of reflection for revalidation purposes.

Most of the tweets were sent during the congress itself. Twitter usage was not maximized by the organizers to update delegates on last-minute changes or to remind participants of parallel sessions or additional sessions, such as sponsored lunchtime meetings. Attendees at the conference used Twitter to take notes from presentations (as illustrated in Figure 4). Because of the small length of tweets, these usually take the form of learning points or salient messages [19-22]. This can be a useful way to remember the little gems of information learned at a congress. Looking at what other people at the congress tweeted or retweeted can further reinforce the “real” learning points from each talk or session [12].

Questions were not posted by any of the Twitter stream contributors to the #WSM12 hashtag probably because of the small numbers involved, but also because systems for posting questions to the presenters had not been arranged by the organizers of the conference nor advertised to the delegates of the meeting in advance. Questions could have been posted by attendees or by people who were not at the meeting who might have questions relating to information posted on tweets. This could have led to a Twitter debate to argue, discuss, seek clarification, and post questions and answers during a presentation. This is usually done on mobile devices during the talk, and is often far less disruptive than whispering to one’s neighbor. The tweets for a particular talk can be collected together and displayed on a Twitter Wall, which is a live display of current tweets for that particular session. Although this can provide a useful means of discussion and posting questions, it can be distracting for speakers and is occasionally subject to abuse [10] and, therefore, not recommended. Perhaps a better option may be to display a list of questions generated on mobile devices during the session at the end of that session to use as a basis for discussion. By viewing posts, asking questions, and joining discussions, Twitter can even allow delegates to participate in parallel sessions at the same time [14]. Discussion points were not used at all in the 2011 AAGBI conference as reported by McKendrick [13]; however, several points of discussion were raised at this conference.

The speakers used Twitter to promote their topic or take-home messages under the #WSM12 hashtag (as illustrated in Figure 4), but no questions were posted by delegates for them to respond to. There was also no feedback from the delegates to the speakers through tweets on their presentation, in which comments about the composition and legibility of slides, for example, could influence future presentations [12]. This is known as self-correction. However, occasionally the opposite can occur with a perpetuation of the original error [7].

Twitter has a maximum time limit of 10 days on its search facility. The tweets can still be found in each individual user’s account, but can no longer be easily grouped together by using the search function. Therefore, it is imperative to create an archive of the Twitter Search results well in advance of this expiry time if one plans to use Twitter as a means of record keeping [23].This method of record keeping was suggested by some of the tweets posted under the #WSM12 hashtag (as illustrated in Figure 4) for the purposes of recollection, continuous professional development [8], and demonstrating reflection during the meeting for revalidation.

Twitter can be further used as a method of amplifying the congress to a wider audience. Tweets posted by delegates can be read by people who were not present at the meeting, who then in turn retweet the message to their followers, creating a second tier of information spreading. This retweeting can continue for many tiers. Some Twitter users have hundreds of thousands of followers and information can be disseminated very quickly over a short span of time.

The tweeters who contributed actively to the Twitter stream in this study presented a potential audience of 3603 people as a first tier of information spreading. A second tier of information spreading, or amplification, was demonstrated in this report by 4 tweeters who did not actively contribute to the Twitter stream, but who retweeted #WSM12 tweets to their 9006 followers. These 4 tweeters only represent a small proportion of the 3603 first-tier followers, the rest of whom potentially could also have retweeted to all their followers creating a “viral” dissemination of the message to a much larger audience than the 9006 demonstrated in this study. However, this assumption presupposes that all a user’s followers are still active on Twitter, and that there is a low rate of redundancy.

Before the conference, in addition to the organizer, 3 other tweeters contributed to the advertising of the congress by retweeting tweets that promoted the conference. These 3 tweeters included the conference venue, a major London publicity organization, and an anesthetic blogger. These 3 organizations had a combined following of 10,894, which provided a significant boost to the advertising power of the organizers and enhanced the promotion and awareness of the conference.

The social element of Twitter could have been better used. Although messages relating to the official conference dinner were posted, no tweets related to lost-and-found items, unofficial social events and dinners, tips for accommodation, or places to go for food and entertainment were posted. In addition, physical meetings could have been arranged with other tweeters at a conference (a “tweetup”).

The final session of the conference should not be seen as the end of that conference’s Twitter stream. This report demonstrates an enhanced use of Twitter after the congress, with 12 tweets being posted compared to 1 tweet in the 2011 AAGBI conference, as reported by McKendrick [13]. The tweets posted displayed the ability to continue discussions previously started at the conference, advertise future meetings, thank attendees, thank colleagues for posting useful information, and illustrating methods of using the Twitter stream for revalidation. To achieve maximum potential from the postconference period, the organizers of the meeting could also have gathered feedback from the delegates, posted interesting statistics, and reflected on various aspects of the conference [14].

Although there are many advantages to Twitter, there are some potential pitfalls. Twitter collects personal information about its users and shares that information with third parties. Third parties can search for characteristics and thereby target users on the basis of their Twitter history and content. Advertisers have even been known to quote users’ tweets in their advertisements [24]. Although Twitter did not initially advertise, in 2010 it introduced a veiled form of advertising called “promoted tweets” [25].

By default, all tweets are made public unless an individual changes the settings or sends the message as a direct message. As an individual posting tweets, it is critically important not to broadcast any information or views on Twitter that might conflict with or defame employers, colleagues, students, academics, researchers, and other University stakeholders [26]. Although it is possible to delete a tweet, often the damage has already been done by that stage. It is essential for those in the public eye to manage their online reputation with great care, and there have been several recent high profile cases paraded in the media for Twitter indiscretions.

Health care professionals have concerns about the use of Twitter that need to be addressed, including patient, personal, and other health care professional’s privacy. Such concerns are increasingly being recognized and discussed [27], and guidance is now being produced by organizations such as the British Medical Association to provide practical and ethical advice to assist doctors [28]. Conference organizers have an obligation to educate participants about Twitter etiquette, protecting their personal identity, and appropriate legal and ethical considerations.

In conclusion, this is the first report to describe the uptake and use of a Twitter stream as an integral part of the communication structure of an anesthetic conference. The usage of Twitter at the 2012 AAGBI Winter Scientific Meeting closely followed trends described for other medical and nonmedical conferences. Therefore, Twitter has potential to be a useful tool at future anesthetic conferences, but there are pitfalls that should be recognized.

## Abbreviations

AAGBI: Association of Anaesthetists of Great Britain and Ireland
SMS: Short Message Service
